# Clinicopathological and prognostic significance of mitogen-activated protein kinases (MAPK) in breast cancers

**DOI:** 10.1007/s10549-016-3967-9

**Published:** 2016-09-03

**Authors:** Dena A. J. Ahmad, Ola H. Negm, M. Layth Alabdullah, Sameer Mirza, Mohamed R. Hamed, Vimla Band, Andrew R. Green, Ian O. Ellis, Emad A. Rakha

**Affiliations:** 1 Division of Cancer and Stem Cells, Department of Histopathology, School of Medicine, Nottingham City Hospital, The University of Nottingham and Nottingham University Hospitals NHS Trust, Nottingham, UK; 2 Department of Pathology, Mosul Medical School, University of Mosul, Mosul, Iraq; 3 School of Medicine, Queen’s Medical Hospital, University of Nottingham, Derby Road, Nottingham, NG7 2UH UK; 4 Faculty of Medicine, Medical Microbiology and Immunology Department, Mansoura University, Mansoura, Egypt; 5 Academic Unit of Clinical Oncology, School of Medicine, Nottingham City Hospital, University of Nottingham, Nottingham, UK; 6 Department of Genetics, Cell Biology and Anatomy, University of Nebraska, Omaha, USA

**Keywords:** MAPK signalling pathways, Reverse phase protein microarray (RPPA), Breast cancer

## Abstract

**Background:**

Mitogen-activated protein kinases (MAPKs) are signalling transduction molecules that have different functions and diverse behaviour in cancer. In breast cancer, MAPK is related to oestrogen receptor (ER) and HER2.

**Methods:**

Protein expression of a large panel of MAPKs (JNK1/2, ERK, p38, C-JUN and ATF2 including phosphorylated forms) were assessed immunohistochemically in a large (*n* = 1400) and well-characterised breast cancer series prepared as tissue microarray. Moreover, reverse phase protein array was applied to quantify protein expression of MAPKs in six breast cancer cell lines with different phenotypes including HER2-transfected cells.

**Results:**

MAPKs expression was associated with clinicopathological variables characteristic of good prognosis. These associations were most significant in the whole series and in the ER+ subgroup compared to other BC classes. Most of MAPKs showed a positive association with ER, BCL2 and better outcome and were negatively associated with the proliferation marker Ki67 and p53. Association of MAPK with HER2 was mainly seen in the ER- subgroup. Reverse phase protein array confirmed immunohistochemistry results and revealed differential expression of MAPK proteins in ER+ and ER− cell lines.

**Conclusions:**

MAPKs are associated with good prognosis and their expression is mainly related to ER. Studying a large panel rather than individual biomarkers may provide improved understanding of the pathway.

**Electronic supplementary material:**

The online version of this article (doi:10.1007/s10549-016-3967-9) contains supplementary material, which is available to authorized users.

## Introduction

Mitogen-activated protein kinases (MAPKs) are evolutionary conserved enzymes which function as signal transduction pathways that regulate fundamental cell activities including gene expression, cellular processes such as growth, proliferation, differentiation, migration and apoptosis. MAPKs’ stimulation starts from stimulation of receptor tyrosine kinase (RTK) by growth factors, cytokines, heat shock, mitogen, osmotic stress or stress factors [1]. Stimulated receptors associate with certain adaptor proteins that in turn enhance the recruitment of guanine nucleotide exchange factors (GEFs) in the cell membrane. The latter can stimulate some small GTPase proteins (H-RAS, N-RAS and K-RAS) which eventually regulate the switch from GDP to GTP and vice versa [2]. GEFs can enhance the formation of RAS GTP which is the active form that can bind to different downstream proteins such as RAF, members of PI3K pathway and RAL-GEF–RAL cascade [3–5]. RAF proteins directly phosphorylate MEK1/2 [6, 7] and can activate ERK1/2 [6] which subsequently can enhance different factors such as transcription factors, kinases and phosphatases [2, 6].

Several groups of MAPK family have been identified, including extracellular regulatory kinase (ERK1/2/5, also known as classical MAPK), p38 MAPK and c-Jun N-terminal kinase/stress-activated protein kinases (JNK1/2/3/SAPKs). Activation of each group requires dual phosphorylation of threonine and tyrosine within the activation loop of the MAPK through a three-tiered cascade composed of MAPK, MAPK kinases (MEKs) and MAPKKK. MEKs are specific for each isoform of MAPK family. For instance, ERK1/ERK2 is activated by MEK1 and MEK2, JNK is activated by MEK4 and MEK7 and p38 MAPK is activated by MEK3 and MEK6 [8, 9].

The function of MAPKs in breast cancer (BC) is complex due to different responses they modulate and their interaction with different pathways [9–11]. MAPKs have been investigated in BC including their interaction with oestrogen receptor (ER) and HER2; however, conflicting results were reported and the exact role of MAPKs in BC and their interaction with ER and HER2 remain to be determined [12–14].

The aim of this study is to investigate the role of MAPK signalling cascade in BC utilising a large panel of biomarker and a large well-characterised series of early stage BC prepared as TMA using IHC. MAPK expression was assessed in the different molecular classes based on expression of ER and HER2 status. In addition, reverse phase protein array (RPPA) was employed to quantify MAPK protein expression levels in different BC cell line phenotypes including the impact of HER2 transfection in the ER+ and ER− cell lines. This retrospective study adheres to REMARK criteria [15].

## Materials and methods

In this study, 1400 cases of unselected operable invasive BC series were included; these are part of the Nottingham Tenovus primary BC series that were enrolled to City Hospital in the period from 1988 to 1998. All patients’ information was available including age (<70 years, mean = 55 years), menopausal status, tumour characteristics (grade, lymph node stage and size), vascular invasion and Nottingham prognostic index (NPI) [16]. The management protocol was uniform and included systemic hormonal therapy for those whose NPI was >3.4, and if they were premenopausal, Zoladex was added. The remaining ER-negative cases received chemotherapy in the form of cyclophosphamide, methotrexate and 5-fluorouracil.

The regular follow-up was collected and included BC-specific survival (time from the surgery until the patient die from or with BC with follow-up period of 15 years. Locoregional recurrence data were also available in this series. In addition, data on a large number of relevant biomarkers were also available as previously described [17–21].

### Immunohistochemistry (IHC)

IHC staining for different MAPK markers was done using 4 µm sections from TMA blocks as published before [22].

Details of primary antibodies and other relevant biomarkers included in this study are available in Online Resource. These biomarkers were used to molecularly characterise the series and further assess the biological function of MAPK activation in BC.

### Scoring of IHC

TMA slides were manually scored using high-resolution digital images (NanoZoomer, Hamamatsu Photonics, Welwyn Garden City, UK) scanned at ×20 magnification, by using a web-based interface (Distiller, Slidepath Ltd., Dublin, Ireland). H-score and percent were used to score the markers. The median scores and X-tile bioinformatics software (version 3.6.1, 2003–2005, Yale University, USA (http://x-tile.software.informer.com) were both used to derive optimal cut-off points for each marker [23].

### In vitro study

The expression of MAPKs and the interaction with ER and HER2 were evaluated in six different molecular classes of BC cell lines which included the following Wild-type MCF-7 (ER+/HER2−), MDA-MB-231 (ER−/HER2−), SKBR3 (ER−/HER2+) and BT474 (ER+/HER2+) and were obtained from the American Type Culture Collection (Manassas, VA, USA). In addition, MCF-7 (ER+) and MDA-231 (ER−) were stably transfected with HER2 gene to assess the impact of HER2 on MAPK activation in BC in relation to ER status [24]. The HER2 transfection has been confirmed by WB (Online Resource).

### Reverse phase protein microarray (RPPA)

RPPA has been established before [25–28]. In this study, lysates from the six used BC cell lines were used to evaluate the expression of MAPK signalling intermediates. All primary antibodies were evaluated for specificity using Western blotting before using in RPPA as described before [22, 25].

### Statistical analysis

The statistical analysis was performed using Statistical Package for Social Sciences SPSS v21. Chi-square test was used to test the associations between categorical data. Kaplan–Meier test was used for univariate analysis. Kruskal–Wallis test was used to test the associations for the results of RPPA. Moreover, Spearman’s rank correlations were used. A two-tailed *p* value of less than 0.05 was considered significant for all the used statistical tests.

## Results

### MAPK expression in BC tissue

In this study, 16 MAPKs members were investigated. Specificity of MAPKs proteins was confirmed by Western blot which revealed specific band for each protein (Online Resource). IHC staining of MAPKs (pan and phosphorylated (p) ERK1/2, pan JNK1/2, p-JNK1/2, pan p38, p-p38, p-ATF2 and p-C-JUN) revealed nuclear expression of phosphorylated proteins except p-ERK1/2 which showed both nuclear and cytoplasmic expression. The total/unphosphorylated forms showed cytoplasmic expression. All MAPKs proteins showed an equivocal expression in normal breast tissue, DCIS and BC tissue included within the TMA cores at varying degrees ranging from negative to strong positivity (Online Resource).

Cut-off of positivity was chosen for each marker to assess its association with other variables. There were positive correlations between different members of MAPKs using continuous data as well as dichotomised variables (Online Resource).

### The association between MAPKs and clinicopathological variables

Expression of MAPK proteins showed positive correlations with clinicopathological features characteristic of good prognosis including lower grade, early stage, smaller tumour size, absent lymphovascular invasion and lower NPI scores (Table 1).

**Table 1:** The associations between MAPKs and clinicopathological variables in breast cancer

	Pan ERK1/2			Nuclear p-ERK1/2			Cytoplasmic-p-ERK1/2		
	Low *N* (%)	High *N* (%)	*p* value	Low *N* (%)	High *N* (%)	*p* value	Low *N* (%)	High *N* (%)	*p* value
Grade									
1	85 (14)	99 (17)	**0.031**	54 (9)	118 (20)	**<0.0001**	52 (10)	119 (20)	**0.001**
2	199 (32)	203 (36)		156 (28)	241 (41)		172 (32)	220 (36)	
3	338 (54)	268 (47)		352 (63)	235 (39)		310 (58)	271 (44)	
Lymph node stage									
1	379 (61)	343 (60)	0.085	343 (61)	118 (20)	NS	313 (58)	384 (63)	NS
2	180 (29)	188 (33)		170 (30)	241 (41)		164 (31)	178 (29)	
3	63 (10)	40 (7)		49 (9)	235 (39)		57 (11)	48 (8)	
Tumour size									
<2 CM	290 (47)	301 (53)	**0.029**	242 (43)	324 (54)	**<0.0001**	222 (42)	336 (55)	**<0.0001**
>2CM	334 (53)	269 (47)		321 (57)	272 (46)		312 (58)	277 (45)	
Lymphovascular invasion									
No	399 (65)	381 (67)		357 (64)	399 (67)		322 (61)	425 (70)	**0.001**
Definite	219 (35)	190 (33)	NS	202 (36)	192 (33)	NS	210 (39)	181(30)	
NPI									
GPG	170 (28)	183 (34)	**0.015**	121 (23)	209 (37)	**<0.0001**	122 (24)	205 (35)	**<0.0001**
MPG	324 (54)	293 (54)		314 (59)	293 (51)		286 (56)	317 (54)	
PPG	106 (18)	66 (12)		101 (19)	70 (12)		104 (20)	64 (11)	

	Pan JNK1/2			p-JNK1/2			Pan p38		
	Low *N* (%)	High *N* (%)	*p* value	Low *N* (%)	High *N* (%)	*p* value	Low *N* (%)	High *N* (%)	*p* value
Grade									
1	100 (17)	104 (19)	NS	26 (11)	131 (16)	**<0.0001**	26 (11)	131 (16)	**0.002**
2	216 (36)	168 (32)		45 (20)	308 (37)		45 (20)	308 (37)	
3	277 (47)	260 (49)		156 (69)	388 (50)		156 (69)	388 (50)	
Lymph node stage									
1	398 (65)	356 (65)	NS	140 (62)	498 (60)	NS	140 (62)	498 (60)	NS
2	169 (8)	150 (27)		66 (29)	260 (32)		66 (29)	260 (32)	
3	46 (7)	42 (8)		21 (9)	69 (8)		21 (9)	69 (8)	
Tumour size									
<2 CM	283 (50)	274 (55)	0.079	93 (40)	427 (52)	**0.003**	93 (40)	427 (52)	NS
>2 CM	286 (50)	223 (45)		137 (60)	401 (48)		137 (60)	401 (48)	
Lymphovascular invasion									
No	376 (67)	344 (69)	NS	153 (67)	540 (66)	NS	153 (67)	540 (66)	NS
Definite	187 (33)	152 (31)		76 (33)	282 (34)		76 (33)	282 (34)	
NPI									
GPG	192 (33)	181 (35)	NS	47 (21)	255 (32)	**0.002**	47 (21)	255 (32)	**0.013**
MPG	303 (53)	266 (52)		132 (60)	435 (55)		132 (60)	435 (55)	
PPG	79 (14)	69 (13)		42 (19)	103 (13)		42 (19)	103 (13)	

	p-p38			p–C-JUN			p-ATF2		
	Low *N* (%)	High *N* (%)	*p* value	Low *N* (%)	High *N* (%)	*p* value	Low *N* (%)	High *N* (%)	*p* value
Grade									
1	120 (13)	82 (21)	**<0.0001**	62 (14)	135 (16)	**<0.0001**	114 (12)	81 (24)	**<0.0001**
2	293 (31)	166 (43)		119 (26)	313 (38)		303 (32)	139 (42)	
3	525 (56)	142 (36)		268 (60)	380 (46)		539 (56)	114 (34)	
Lymph node stage									
1	550 (59)	261 (67)	**0.008**	266 (59)	517 (62)	NS	571 (60)	208 (62)	NS
2	310 (33)	96 (24)		143 (32)	251 (30)		311 (32)	92 (28)	
3	78 (8)	34 (9)		40 (9)	61 (8)		74 (8)	35 (10)	
Tumour size									
<2 CM	423 (45)	225 (58)	**<0.0001**	206 (46)	421 (51)	0.080	451 (47)	173 (52)	NS
>2 CM	517 (55)	166 (42)		245 (54)	408 (49)		507 (53)	162 (48)	
Lymphovascular invasion									
No	602 (65)	284 (73)	**0.002**	278 (62)	566 (69)	**0.015**	611 (64)	234 (71)	**0.032**
Definite	332 (35)	104 (27)		171 (38)	258 (31)		344 (36)	98 (29)	
NPI									
GPG	235 (26)	159 (43)	**<0.0001**	114 (26)	264 (33)	**0.001**	240 (26)	132 (41)	**<0.0001**
MPG	508 (57)	177 (47)		238 (55)	426 (54)		530 (58)	143 (45)	
PPG	156 (17)	38 (10)		84 (19)	100 (13)		149 (16)	44 (14)	

*p* values in bold denote significant ones (<0.050), borderline: (0.05–0.09), NS: >0.09) and the same in all tables

*NPI* Nottingham prognostic index, *GPG* good prognostic group, *MPG* moderate prognostic group, *PPG* poor prognostic group

### The association between MAPKs and key BC biomarkers

There was significant correlation with ER and HER2 in addition to other key BC biomarkers including the proliferation marker KI67-LI and the apoptosis markers BCL2 and p53 (Table 2). Pan ERK1/2 showed strong positive association with ER and BCL2 but only showed borderline negative association with KI67-LI and p53. p-ERK1/2 was positively associated with ER and negatively with BCL2 but only its nuclear form showed a positive association with BCL2 and a negative association with HER2 and p53. Pan JNK1/2 was associated with downregulation of ER and BCL2; however, its phosphorylated form was associated with increased expression of ER, BCL2 and with downregulation of KI67-LI. p-p38 and its total form were positively associated with ER and BCL2 and negatively with KI67-LI.

**Table 2:** Associations between MAPKs and biological markers in the whole series

	Pan ERK1/2			Nuclear p-ERK1/2			Cytoplasmic-p-ERK1/2		
	Low *N* (%)	High *N* (%)	*p* value	Low *N* (%)	High *N* (%)	*p* value	Low *N* (%)	High *N* (%)	*p* value
ER									
Negative	176 (28)	112 (20)	**<0.0001**	186 (33)	102 (17)	**<0.0001**	146 (28)	139 (23)	**0.066**
Positive	444 (72)	460 (80)		373 (67)	487 (83)		385 (72)	468 (77)	
HER2									
Negative	516 (87)	480 (87)	NS	443 (83)	516 (90)	**<0.0001**	442 (86)	506 (86)	NS
Positive	75 (13)	70 (13)		93 (17)	58 (10)		71 (14)	79 (14)	
KI67-LI									
Low	200 (39)	208 (46)	0.050	153 (34)	224 (47)	**<0.0001**	159 (37)	233 (47)	**0.003**
High	307 (61)	247 (54)		304 (66)	250 (53)		266 (63)	263 (53)	
P53									
Negative	400 (68)	405 (73)	0.064	361 (68)	416 (74)	**0.032**	351 (69)	416 (72)	NS
Positive	185 (32)	147 (27)		172 (32)	149 (26)		160 (31)	158 (28)	
BCL2									
Negative	199 (47)	134 (32)	**<0.0001**	113 (37)	77 (24)	**<0.0001**	174 (46)	183 (41)	NS
Positive	225 (53)	283 (68)		196 (63)	239 (76)		208 (54)	269 (59)	

	Pan JNK1/2			p-JNK1/2			Pan p38		
	Low *N* (%)	High *N* (%)	*p* value	Low *N* (%)	High *N* (%)	*p* value	Low *N* (%)	High *N* (%)	*p* value
ER									
Negative	130 (22)	149 (28)	**0.015**	94 (41)	189 (23)	**<0.0001**	221 (31)	89 (19)	**<0.0001**
Positive	466 (78)	382 (72)		136 (59)	633 (77)		492 (69)	387 (81)	
HER2									
Negative	478 (88)	406 (85)	0.092	184 (83)	690 (87)	NS	594 (88)	385 (87)	NS
Positive	63 (12)	73 (15)		37 (17)	103 (13)		81 (12)	59 (13)	
KI67-LI									
Low	199 (44)	172 (45)	NS	58 (31)	292 (44)	**0.001**	594 (88)	385 (87)	0.058
High	255 (56)	213 (55)		127 (69)	368 (56)		81 (12)	59 (13)	
P53									
Negative	394 (74)	331 (69)	NS	149 (68)	563 (72)	NS	460 (69)	331 (74)	0.060
Positive	141 (26)	149 (31)		70 (32)	222 (28)		205 (31)	113 (26)	
BCL2									
Negative	150 (38)	183 (48)	**0.004**	90 (52)	247 (41)	**0.005**	224 (46)	114 (34)	**0.001**
Positive	246 (62)	197 (52)		82 (48)	368 (59)		259 (54)	221 (66)	

	p-p38			p–C-JUN			p-ATF2		
	Low *N* (%)	High *N* (%)	*p* value	Low *N* (%)	High *N* (%)	*p* value	Low *N* (%)	High *N* (%)	*p* value
ER									
Negative	246 (26)	70 (18)	**0.002**	130 (29)	178 (22)	**0.005**	260 (27)	51 (15)	**0.003**
Positive	692 (74)	317 (82)		321 (71)	645 (78)		693 (73)	281 (85)	
HER2									
Negative	777 (86)	329 (89)	NS	371 (86.3)	687 (86.5)	NS	777 (85)	299 (92)	**0.001**
Positive	128 (14)	42 (11)		59 (13.7)	107 (13.5)		138 (15)	25 (8)	
KI67-LI									
Low	292 (39)	161 (53)	**<0.0001**	127 (35.6)	304 (45.8)	**0.002**	289 (38)	144 (53)	**<0.0001**
High	463 (61)	142 (47)		230 (64.4)	360 (54.2)		466 (62)	130 (47)	
P53									
Negative	637 (70)	267 (73)	NS	302 (70.2)	564 (71.5)	NS	634 (70)	243 (76)	**0.025**
Positive	269 (30)	97 (27)		128 (29.8)	225 (28.5)		277 (30)	76 (24)	
BCL2									
Negative	297 (43)	99 (36)	**0.037**	138 (43.5)	249 (41.2)	NS	305 (44)	84 (34)	**0.009**
Positive	391 (57)	177 (64)		179 (56.5)	355 (58.8)		396 (56)	163 (66)	

p-ATF2 and p-C-JUN, which are downstream markers of the MAPK pathway, showed positive associations with ER and negative association with KI67-LI. p-ATF2 also showed positive association with BCL2 and negative with HER2 and p53.

Within ER+ tumours, most of the associations observed in the whole series remained significant including nuclear p-ERK1/2, p-p38 and p-ATF2 (Table 3). When the ER+ group was further stratified based on HER2 expression, some associations were maintained in the ER+/HER2− subgroup (Online Resource) but not in the ER+/HER2+ tumours. Interestingly, when the analysis was restricted to HER2− tumours, the associations observed in the whole cohort and in the ER+ class were maintained (Online Resource). Importantly, when the analysis was restricted to ER− class, pan-ERK1/2 and p-p38 were associated positively with HER2 (*p* = 0.006, 0.003 respectively; Online Resource).

**Table 3:** Associations between MAPK and biological markers in ER-positive tumours

	Pan ERK1/2			N-p-ERK1/2			C-p-ERK1/2		
	Low *N* (%)	High *N* (%)	*p* value	Low *N* (%)	High *N* (%)	*p* value	Low *N* (%)	High *N* (%)	*p* value
HER2									
Negative	379 (89)	407 (92)	NS	315 (87)	440 (93)	**0.005**	337 (90)	409 (91)	NS
Positive	46 (11)	37 (8)		47 (13)	34 (7)		38 (10)	42 (9)	
KI67-LI									
Low	179 (50)	190 (52)	NS	137 (45)	216 (55)	**0.005**	141 (46)	211 (55)	**0.010**
High	180 (50)	174 (4.8)		171 (55)	175 (45)		169 (54)	170 (45)	
P53									
Negative	325 (77)	364 (82)	0.093	282 (79)	368 (79)	NS	291 (78)	351 (79)	NS
Positive	96 (23)	81 (18)		76 (21)	99 (21)		81 (22)	92 (21)	
BCL2									
Negative	101 (33)	81 (23)	**0.005**	105 (36)	87 (25)	**0.001**	95 (33)	95 (27)	NS
Positive	206 (67)	269 (77)		186 (64)	267 (75)		191 (67)	254 (73)	

	Pan JNK1/2			p-JNK1/2			Pan p38		
	Low *N* (%)	High *N* (%)	*p* value	Low *N* (%)	High *N* (%)	*p* value	Low *N* (%)	High *N* (%)	*p* value
HER2									
Negative	386 (91)	313 (90)	NS	115 (87)	553 (91)	NS	434 (92)	328 (90)	NS
Positive	36 (9)	33 (10)		17 (13)	57 (9)		35 (8)	36 (10)	
KI67-LI									
Low	185 (53)	152 (54)	NS	48 (44)	265 (53)	0.098	208 (53)	158 (52)	NS
High	164 (47)	127 (46)		61 (56)	237 (47)		187(47)	144 (48)	
P53									
Negative	338 (80)	267 (7.8)	NS	108 (83)	475 (78)	NS	373 (81)	295 (81)	NS
Positive	83 (20)	76 (22)		22 (17)	131 (22)		88 (19)	69 (19)	
BCL2									
Negative	85 (27)	84 (31)	NS	36 (33)	131 (28)	NS	105 (31)	66 (24)	0.057
Positive	231 (73)	186 (69)		73 (67)	342 (72)		237 (69)	211 (76)	

	p-p38			p–C-JUN			p-ATF2		
	Low *N* (%)	High *N* (%)	*p* value	Low *N* (%)	High *N* (%)	*p* value	Low *N* (%)	High *N* (%)	*p* value
HER2									
Negative	593 (88)	283 (95)	**0.001**	273 (89)	563 (90)	NS	594 (89)	256 (94)	**0.010**
Positive	78 (12)	15 (5)		32 (11)	59 (10)		73 (11)	15 (6)	
KI67-LI									
Low	264 (47)	147 (60)	**0.001**	113 (44)	277 (54)	**0.011**	256 (47)	136 (58)	**0.003**
High	295 (53)	97 (40)		143 (56)	237 (46)		292 (53)	97 (42)	
P53									
Negative	533 (80)	236 (79)	NS	248 (81)	488 (79)	NS	523 (79)	523 (79)	NS
Positive	135 (20)	61 (21)		58 (19)	128 (21)		140 (21)	140 (21)	
BCL2									
Negative	162 (31)	50 (23)	**0.026**	71 (30)	136 (29)	NS	153 (29)	55 (27)	NS
Positive	360 (69)	168 (77)		163 (70)	333 (71)		368 (71)	151 (73)	

### Outcome analysis

**Fig. 1:**
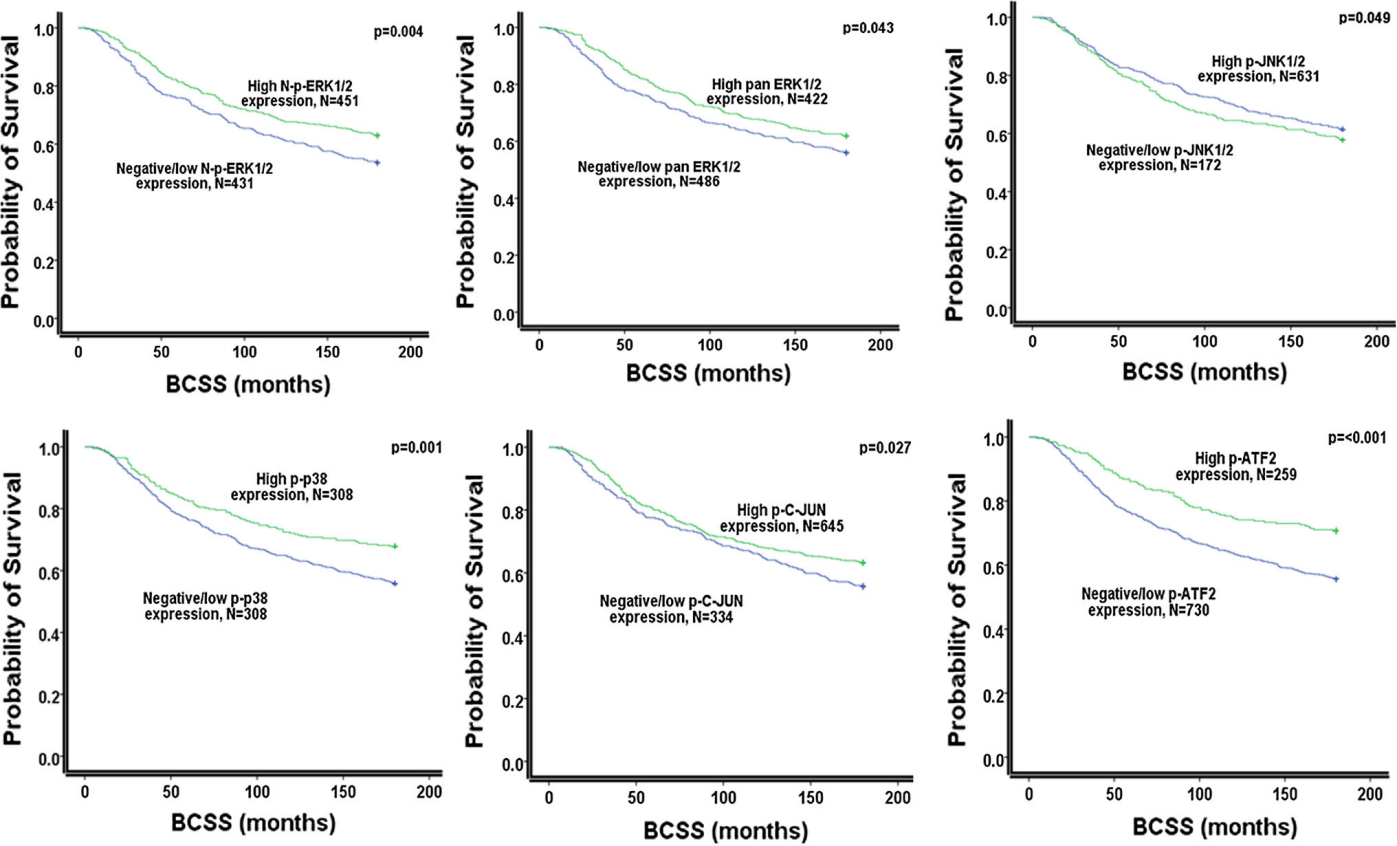
This Kaplan–Meier survival curve illustrates that MAPK members including the nuclear p-ERK1/2, its total form ERK1/2, p-JNK1/2, p-p38 and the two transcription factors p-C-JUN and p-ATF2 are all associated significantly with prolonged BCSS in the whole series

**Fig. 2:**
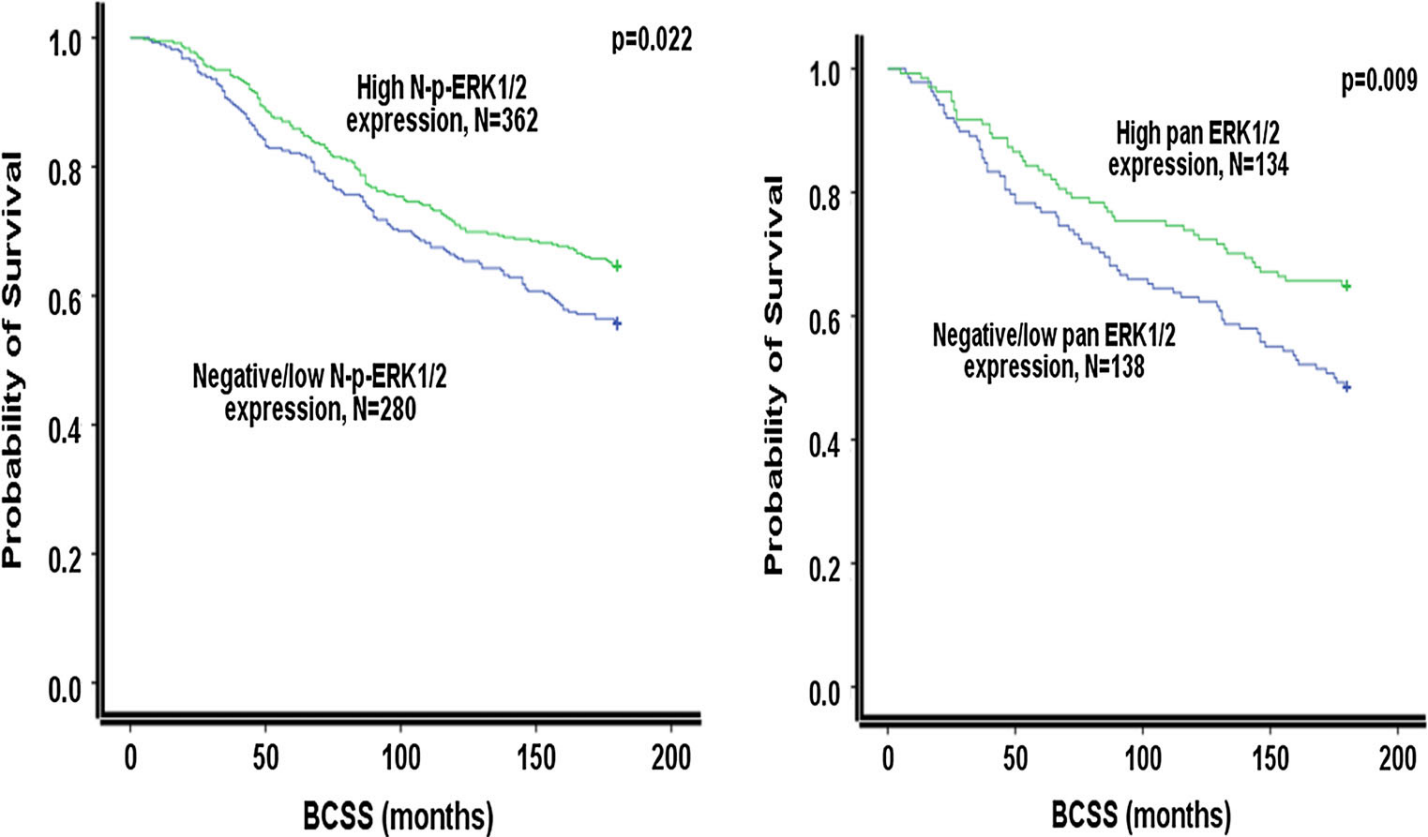
Kaplan–Meier survival curve showing the associations of MAPKs (nuclear (N)p ERK1/2 and pan ERK1/2 are associated significantly with prolonged BCSS in ER-positive series

Univariate survival analyses revealed that MAPKs (pan ERK1/2, nuclear p-ERK1/2, p-JNK1/2, p-p38, p-C-JUN and p-ATF2) were associated with better outcome in terms of prolonged BCSS (Fig. 1). ERK1/2 and N-p-ERK1/2 showed better associations with prolong survival within ER+BC (Fig. 2).

### Proteomic analysis of BC cell lines

RPPA was consistent with IHC results and confirmed that MAPK expression was higher in ER+ compared to ER− cell lines and in the ER+/HER2− cell lines compared to ER+/HER2+. In the ER− cell lines, there was a trend towards positive correlation between MAPs and HER2 expression. Interestingly, most of these MAPKs showed higher expression in ER+HER2− compared to ER−HER2− cell line.

**Fig. 3:**
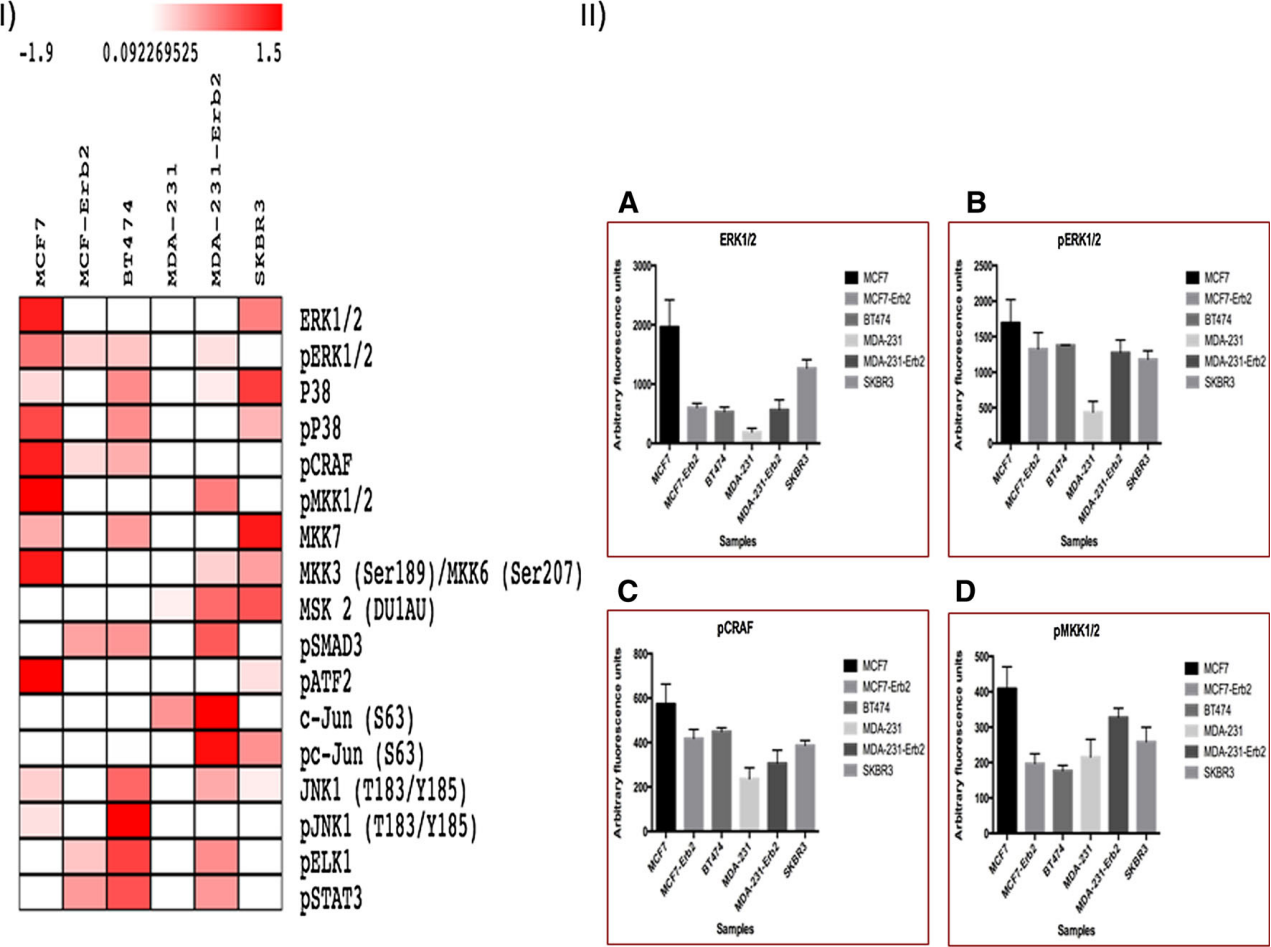
Heat-map showing different MAPK pathway intermediates studied in six different breast cancer cell lines. *Rows* represent the different signalling molecules studied. *White* and *red* denote markers that are present at lower and higher levels, respectively. II: *Bar graphs* representing measurement of selected target proteins from the heat-map data, including **a** ERK1/2, **b** pERK1/2, **c** pCRAF and **d** pMKK1/2. Data are shown as individual sample data (mean of four technical replicates per sample) of three different experiments. Signal represents arbitrary fluorescence units; AFU, after background subtraction and normalisation to β-actin. Significance values were derived using the Kruskal–Wallis test

For example, there was a significant increase in the expression of p-C-RAF in ER+HER2− (MCF-7) cell line compared to ER+HER2+ transfected (T) while in the ER− cell lines there was high expression of this protein in ER−HER2+ cell line compared to ER-HER2− and the difference between these two cell lines was statistically significant. Regarding MEK1/2, which is the downstream mediator of p-C-RAF, the same association was noticed. Similarly, ERK1/2 which is the downstream mediator of MEK1/2, showed an increase in its expression in ER+HER2− compared to ER+HER2+ and high expression in ER-HER2+ (W and T) compared to ER−HER2− cell lines (Fig. 3). The details for the expression of all the MAPK markers are shown in (Online Resource), with all the *p* values for the comparison of the expression levels between the different cell lines.

## Discussion

Several studies have emphasised the role of MAPKs in cancer progression [13, 29–31]. The functions of MAPKs in BC appear to be complex owing to several cellular responses that they modulate and their interaction with different pathways including the key BC genes ER and HER2. In the current study, the role of MAPKs in BC and how the expression of ER and HER2 might influence their function were investigated using a large panel of MAPK proteins and the results were validated in vitro using RPPA and different BC cell lines. The results showed that generally most of MAPKs are associated with good prognostic features in the whole series and in the ER+ tumours. MAPKs are mainly related to ER expression and this finding was observed using IHC and validated by RPPA.

Our results are consistent with others [32] who demonstrated that ERK1/2 and p-ERK1/2 were associated with good clinicopathological variables and that by Hsu et al. [33] who found that p-ERK1/2 is required for inducing apoptosis especially in MCF-7 and MDA-231 cell lines. The association between MAPKs and good prognostic variable may be related to their roles in inducing apoptosis. p-JNK1/2 also has been shown to be stimulated by stress or growth factors and either one can enhance p-JNK1/2 to stimulate apoptosis [34] and even p-JNK1/2 augments cell death signalling in slowly growing MCF-7 cells under the influence of high estradiol level [35]. Interestingly, a drug “pseudolaric acid B” whose function mimics the upstream mediators of MAPKs was used in an in vitro experiment and this revealed the apoptotic function of JNK and its phosphorylated form upon activation [36]. Moreover, several studies have demonstrated the role of p-p38/MAPK in inducing apoptosis in BC and some suggested that this effect is mediated by TGF-β [37–40]. p-ATF2 is thought to have dual functions independent of each other; the first is a tumour suppressor protein and the other function is related to DNA damage response pathway [41] and importantly, the former function has been confirmed by different studies [38, 42, 43]. The above results regarding the association between MAPKs and apoptosis were supported by our findings that MAPKs were positively associated with ER, BCL2 and negatively with HER2 (or no association with some of them), KI67-LI and p53.

Consistent with previous studies that assessed the prognostic value of individual MAPK members [32, 43, 44], in this study we identified an association between MAPKs proteins and better outcome in terms of longer survival time. Importantly, in the current research, pan ERK1/2 and p-ERK1/2 showed an association with better outcome in patients with ER+ tumours who are candidate for endocrine therapy. Consistent with this, Busch et al. [45] have demonstrated that high p-ERK1/2 was an independent predictor of better outcome in tamoxifen-treated patients. Thus, this study implies that MAPKs could be prospective surrogate biomarkers in assessing benefit from endocrine therapy.

In the current study, RPPA results were consistent with IHC findings for those proteins used in both techniques and the findings were also consistent for those MAPK members that were used only in RPPA. In the ER−/HER2+ cell line and in line with our results, a previous study demonstrated that activation of MAPK is associated with loss of ER phenotype especially in those overexpressing HER2 [46]. Ostrakhovitch et al. [47] have found that in MDA-231 cell line where p53 is mutated or suppressed, the phosphorylation of ERK1/2 was strong. Meanwhile, p-p38 overexpression in ER- tumours is thought to be associated with proliferation and progression of cancer. This is attributed to the fact that p-p38 can mediate proliferation only in BC cells that express mutant p53 and not the wild one, a finding commonly seen in ER− tumours rather than ER+ tumours [48]. Interestingly, Creighton et al. [49] indicated that hyperactivation of MAPK leads to the loss of ER expression and plays a role in the generation of the ER-negative phenotype. Although our study supports the differential expression of MAPKs between ER+ and ER− phenotypes, it indicates that MAPKs play an important role in ER+ tumours and that their expression in ER+ tumours is associated with better outcome. Supporting this, Atanaskova et al. [50] have indicated that activation of ER by MAPK enhances the expression of ER-regulated genes, accelerates tumour growth, and that MAPK/ER crosstalk enhances ER−mediated signalling without diminishing sensitivity to the inhibitory effects of anti-estrogens. However, in the ER-negative subgroup, it has been reported that the oncogenic effect of MAPKs is related to HER2 overexpression [49], which further support our findings.

The role of MAPKs in enhancing cell cycle arrest is also encountered. This has been attributed to the fact that innate tumour suppressor mechanisms are activated in response to aberrant oncogenic stimulation and remarkably induce growth inhibition that is referred to as oncogenic-induced senescence [51, 52]. In addition, accumulating evidence has demonstrated that sequestration of cytoplasmic ERK in the cytoplasm induced by proapoptotic molecules such as death-associated protein kinase could ultimately augment the apoptotic action of these proapoptotic proteins [53]. In this regard, a study revealed that feedback inhibitory signals are common from mutated RAS and RAF to the upstream mediators, which will omit further stimulation through this pathway being thoughtfully mediated by HDM2 and FOXO3 [54]. The previous findings might explain why high expression of MAPKs in ER+ tumours was associated with good features and outcome in our IHC findings and why the function is different in ER + and ER− cell lines by using RPPA. In addition, another reason which can explain the dual behaviour of these MAPKs is that they function in a cell context-specific and cell type-specific way to mediate signals that can lead to diverse cellular functions. Furthermore, the function of these MAPKs is influenced by their crosstalk and interaction with other pathways which can influence their behaviour [9–11].

Despite the limitations of this study and type of techniques employed, the findings of this study collectively together with other previous publications mentioned above support the tumour suppressor role of MAPK kinase pathway in BC and that active pathway is associated with variables of good prognosis and better outcome. In addition, our results provide further evidence that ER is the main player related to the function of MAPK in BC; however, when HER2 is overexpressed, the role of ER becomes less significant and there is some evidence that HER2 can play a major role in controlling the MAPK pathway activation in ER-negative tumours.

In conclusion, our study illustrated the role of MAPKs and their signalling in BC and demonstrated that MAPKs are associated with good prognostic features and show differential expression within ER+ and ER− groups.

## Electronic Online Resource

Below is the link to the Online Resource.
Online Resource 1 (DOCX 2121 kb)
