# Supplementary material for: Clinicopathological and prognostic significance of mitogen-activated protein kinases (MAPK) in breast cancers
Source: Breast Cancer Res Treat. 2016 Sep 3;159(3):457–67. doi: 10.1007/s10549-016-3967-9 (PMC5021722; doi:10.1007/s10549-016-3967-9)
Supplement: Supplementary file 1 — Online Resource 1 (DOCX 2121 kb) [file 10549_2016_3967_MOESM1_ESM.docx]

**Mitogen activated protein kinase signalling proteins are associated with good prognosis in oestrogen receptor positive breast cancer**

Breast Cancer Research and Treatment

Dena A. J Ahmad, Ola H Negm, M. Layth Alabdullah, Sameer Mirza, Mohamed R Hamed, Vimla Band, Andrew R Green, Ian O Ellis and Emad A Rakha

**Corresponding author:**

**Dr Ola H Negm**

School of Medicine, Queen’s Medical Hospital, University of Nottingham

Derby Road, NG7 2UH, Nottingham, UK.

**Email:** [**ola.negm@nottingham.ac.uk**](mailto:ola.negm@nottingham.ac.uk)

**Conflict of interest:** The authors have declared that no competing interests exist.

Table 1: The characteristics of the antibodies used in this studies for different purposes

| **Antibody** | **Species** | **Molecular weight( KDa)** | **Incubation (IHC)** | **IHC localisation** | **Negative (IHC)**  **N (%)** | **Positive (IHC)**  **N (%)** | **Positive Cut-Offs** | **Method** | **Purpose** |
| --- | --- | --- | --- | --- | --- | --- | --- | --- | --- |
| **p-c-RAF** | rabbit | 74 | - | - | - | - | - | - | WB,RPPA |
| **p-MEK1/2** | rabbit | 45 | - | - | - | - | - | - | WB,RPPA |
| **p-MKK3(ser189/6(ser207)** | rabbit | 40 | - | - | - | - | - | - | WB,RPPA |
| **p-MKK7(S217/221)** | rabbit | 48 | - | - | - | - | - | - | WB,RPPA |
| **ERK1/2** | rabbit | 42,44 | 1 hour | Cytoplasmic | 625 (52.3) | 571 (47.7) | >100(H-score) | X-tile | IHC,WB,RPPA |
| **p-ERK1/2 (Pt185/pY187)** | rabbit | 42,44 | overnight | Nuclear: | 561 (48.4) | 597 (51.6) | >140 (H-score) | X-tile | IHC,WB,RPPA |
|  |  |  |  | Cytoplasmic: | 533 (46.5) | 613 (53.5) | >30(H-score) | X-tile | IHC,WB,RPPA |
| **JNK1/2** | mouse | 46,54 | 1 hour | Cytoplasmic: | 573 (53.3%) | 502 (46.7%) | >103 (H-score) | X-tile | IHC,WB,RPPA |
| **p-JNK1/2(T183/Y185)** | rabbit | 46,54 | overnight | Nuclear | 229 (21.7%) | 827 (78.3%) | >124 (H-score) | X-tile | IHC,WB,RPPA |
| **P38** | rabbit | 38 | 1 hour | Cytoplasmic | 703 (60.3%) | 463 (39.7%) | >112 (H-score) | X-tile | IHC,WB,RPPA |
| **p-p38 (T180/Y182)** | rabbit | 43 | overnight | Nuclear: | 941 (70.5%) | 394 (29.5%) | >110 (H-score) | X-tile | IHC,WB,RPPA |
| **p-ATF2 (Thr69/71)** | rabbit | 70 | overnight | Nuclear: | 958 (73.9%) | 338 (26.1%) | >70 (H-score) | X-tile | IHC,WB,RPPA |
| **C-JUN** | rabbit | 48 |  |  |  |  |  |  | WB,RPPA |
| **p-C-JNU (Ser 73)** | rabbit | 48 | overnight | Nuclear: | 453 (35.3%) | 831 (64.7%) | >3 (percent) | X-tile | IHC,WB,RPPA |
| **P-SMAD3** | rabbit | 52 | - | - | - | - | - | - | WB,RPPA |
| **p-MSK2** | rabbit | 85 | - | - | - | - | - | - | WB,RPPA |
| **p-ELK1** | rabbit | 47 | - | - | - | - | - | WB,RPPA | WB,RPPA |
| Some of the antibodies in this study were used for IHC purpose. The specificity of all antibodies used in this study was checked using WB by using a mixture of cell lysate. The expression of all antibodies used in this study was tested using RPPA technique. | | | | | | | | | |

Table 2 : Details of the antibodies used for comparison in this study.

| **Marker** | **Name** | **Dilution** | **Cut-Offs** | **Supplier** | **Pre Treatment** |
| --- | --- | --- | --- | --- | --- |
| **1-Hormone receptors (HRs)** | ER [clone 1D5]  PgR[clone PgR 636]  AR[clone F39.4.1] | 1:150  1:100  1:30 | 0  0  0 | DAKO  DAKO  Biogenex | Microwave  No  No |
| **2-luminal cytokeratins (CKs)** | Ck7/8 [clone CAM 5.2]  Ck18 [clone DC10]  Ck19 [clone BCK 108] | 1:2  1:50  1:100 | 50*  50*  50* | Becton Dickinson  DAKO  DAKO | Microwave |
| **4-Other ER related proteins &**  **ER co-regulators** | Trefoil factor 3(TFF3 )  Trefoil factor 1(TFF1)  Forkhead box protein A1 (FOXA1) [clone 2F83]  Trans-acting T-cell-specific transcription factor (GATA3) [HG3-31] | 3µg/ml  1:2000  1:2,000  1:80  1:30 | 100*  100*  10  60 | Abcam  Abcam  Abcam  Santa Cruz Biotechnology | Microwave  Microwave  Microwave  No |
| **5- Proliferation, apoptosis related markers And tumour suppressor proteins** | Ki-67[clone MIB1]  Bcl2 (pro-apoptotic) [clone 124]  p53 [clone DO7] | 1:100  1:100  1:50 | 13  30  5 | DAKO  DAKO  Novocastra | No  No  Microwave |
| **7-HER family proteins** | HER2 [clone cerbB-2] | 1:250 | 10 | DAKO | Microwave |
| - Flagged numbers are H-score values and the non-flagged ones are chosen from percentages of expressed cells. | | | | | |

Table 3: Spearmann’s Rank correlation of the different MAPK proteins used in this study

|  | | | | | | | | | | |
| --- | --- | --- | --- | --- | --- | --- | --- | --- | --- | --- |
|  | | **p-JNK1/2** | **JNK1/2** | **N-p-ERK1/2** | **C-p-ERK1/2** | **ERK1/2** | **p38** | **N-p_p38** | **p-c-jun** | **p_ATF2** |
| *^1^p-JNK1/2 | **Spearmann’s Rank correlation** | * | .074 | .583 | .318 | .317 | .111 | .433 | .230 | .409 |
|  | **p-value** |  | **.033** | **.000** | **.000** | **.000** | **.001** | **.000** | **.000** | **.000** |
|  | **Number of cases** |  | 627 | 922 | 914 | 914 | 733 | 866 | 830 | 846 |
| JNK1/2 | **Spearmann’s Rank correlation** | .074 | * | .097 | .103 | .124 | .119 | .094 | .108 | .075 |
|  | **p-value** | **.033** |  | **.006** | **.004** | **.001** | **.000** | **.004** | **.002** | **.021** |
|  | **Number of cases** | 627 |  | 672 | 662 | 662 | 769 | 781 | 750 | 743 |
| *^2^N-p-ERK1/2 | **Spearmann’s Rank correlation** | .583 | .097 | * | .640 | .556 | .185 | .528 | .370 | .501 |
|  | **p-value** | **.000** | **.006** |  | **.000** | **.000** | **.000** | **.000** | **.000** | **.000** |
|  | **Number of cases** | 922 | 672 |  | 1148 | 1148 | 819 | 960 | 926 | 941 |
| *^3^C-p-ERK1/2 | **Spearmann’s Rank correlation** | .318 | .103 | .640 | * | .726 | .124 | .400 | .360 | .323 |
|  | **p-value** | **.000** | **.004** | **.000** |  | **.000** | **.000** | **.000** | **.000** | **.000** |
|  | **Number of cases** | 914 | 662 | 1148 |  | 1149 | 809 | 953 | 919 | 934 |
| ERK1/2 | **Spearmann’s Rank correlation** | .317 | .124 | .556 | .726 | * | .114 | .340 | .263 | .268 |
|  | **p-value** | **.000** | **.001** | **.000** | **.000** |  | **.001** | **.000** | **.000** | **.000** |
|  | **Number of cases** | 914 | 662 | 1148 | 1149 |  | 809 | 953 | 919 | 934 |
| p38 | **Spearmann’s Rank correlation** | .111 | .119 | .185 | .124 | .114 | * | .113 | .147 | .125 |
|  | **p-value** | **.001** | **.000** | **.000** | **.000** | **.001** |  | **.000** | **.000** | **.000** |
|  | **Number of cases** | 733 | 769 | 819 | 809 | 809 |  | 919 | 887 | 907 |
| N-p_p38 | **Spearmann’s Rank correlation** | .433 | .094 | .528 | .400 | .340 | .113 | * | .372 | .535 |
|  | **p-value** | **.000** | **.004** | **.000** | **.000** | **.000** | **.000** |  | **.000** | **.000** |
|  | **Number of cases** | 866 | 781 | 960 | 953 | 953 | 919 |  | 1227 | 1235 |
| p_c-jun | **Spearmann’s Rank correlation** | .230 | .108 | .370 | .360 | .263 | .147 | .372 | * | .372 |
|  | **p-value** | **.000** | **.002** | **.000** | **.000** | **.000** | **.000** | **.000** |  | **.000** |
|  | **Number of cases** | 830 | 750 | 926 | 919 | 919 | 887 | 1227 |  | 1180 |
| p_ATF2 | **Spearmann’s Rank correlation** | .409 | .075 | .501 | .323 | .268 | .125 | .535 | .372 | * |
|  | **p-value** | **.000** | **.021** | **.000** | **.000** | **.000** | **.000** | **.000** | **.000** |  |
|  | **Number of cases** | 846 | 743 | 941 | 934 | 934 | 907 | 1235 | 1180 |  |
| ***Flagged areas represent the analysis of each marker with itself. *^1^ p= phosphorylated, *^2^N=nuclear, and *^3^C-cytoplasmic., p values significant in bold.** | | | | | | | | | | |

Table 4: The associations between MAPK and biological markers in ER+HER2- tumours

|  | **Pan ERK1/2** | | | **N-p-ERK1/2** | | | **C-p-ERK1/2** | | |
| --- | --- | --- | --- | --- | --- | --- | --- | --- | --- |
|  | Low  N (%) | High  N (%) | *p*-value | Low  N (%) | High  N (%) | *p*-value | Low  N (%) | High  N (%) | *p*-value |
| **KI67-LI**  Low  High | 166(60)  148(47) | 184(56)  145(44) | NS | 119(45)  143(55) | 204(58)  150(42) | **0.003** | 125(46)  148(54) | 197(59)  139(41) | **0.003** |
| **P53**  Negative  Positive | 295(80)  75(20) | 340(84)  63(16) | 0.093 | 250(82)  56(18) | 347(80)  84(20) | NS | 267(81)  63(19) | 323(81)  75(19) | NS |
| **BCL2**  Negative  Positive | 74(28)  190(72) | 64(21)  248(79) | **0.035** | 36(19)  154(81) | 29(12)  206(88) | 0.060 | 38(19)  162(81) | 25(12)  193(88) | NS |
|  | **Pan JNK1/2** | | | **p-JNK1/2** | | | **Pan p38** | | |
|  | Low  N (%) | High  N (%) | *p*-value | Low  N (%) | High  N (%) | *p*-value | Low  N (%) | High  N (%) | *p*-value |
| **KI67-LI**  Low  High | 168(55)  139(45) | 141(57)  106(43) | NS | 44(47)  49(53) | 253(57)  194(43) | NS | 194(54)  164(46) | 152(57)  114(43) | NS |
| **P53**  Negative  Positive | 308(82)  66(18) | 247(81)  57(19) | NS | 95(85)  17(15) | 437(80)  107(20) | NS | 348(82)  75(18) | 274(85)  50(15) | NS |
| **BCL2**  Negative  Positive | 68(24)  216(76) | 64(28)  169(72) | NS | 27(29)  65(71) | 102(25)  312(75) | NS | 85(27)  225(73) | 50(21)  190(79) | 0.075 |
|  | **p-p38** | | | **p-C-JUN** | | | **p-ATF2** | | |
|  | Low  N (%) | High  N (%) | *p*-value | Low  N (%) | High  N (%) | *p*-value | Low  N (%) | High  N (%) | *p*-value |
| **KI67-LI**  Low  High | 246(50)  243(50) | 139(63)  80(37) | **0.001** | 102(46)  122(54) | 262(58)  191(42) | **0.003** | 237(50)  242(50) | 130(61)  83(39) | **0.005** |
| **P53**  Negative  Positive | 480(82)  102(18) | 226(82)  50(18) | NS | 221(83)  46(17) | 454(82)  98(18) | NS | 475(82)  105(18) | 207(82)  45(18) | NS |
| **BCL2**  Negative  Positive | 126(28)  319(72) | 39(20)  160(80) | **0.019** | 53(26)  151(74) | 108(27)  300(73) | NS | 113(25)  334(75) | 49(26)  139(74) | NS |

Table 5: Association between MAPKs and key biomarkers in the HER2- subgroup

|  | **Pan ERK1/2** | | | **N-p-ERK1/2** | | | **C-p-ERK1/2** | | |
| --- | --- | --- | --- | --- | --- | --- | --- | --- | --- |
|  | **Neg/low** | **High** | **p-value** | **Neg/low** | **High** | **p-value** | **Neg/low** | **Positive** | **p-value** |
| **ER**  **Negative**  **Positive** | 134(26.1%)  379(73.9%) | 69(14.5%)  407(85.5%) | **<0.001** | 126(28.6%)  315(71.4%) | 71(13.9%)  440(86.1%) | **<0.001** | 103(23.4%)  337(76.6%) | 92(18.4%)  409(81.6%) | 0.057 |
| **KI67-LI**  **Low**  **High** | 180(42.3%)  246(57.7%) | 192(49.5%)  196(50.5%) | **0.039** | 137(37.3%)  230(62.7%) | 219(52.9%)  195(47.1%) | **<0.001** | 139(39.0%)  217(61.0%) | 216(51.8%)  201(48.2%) | **<0.001** |
| **P53**  **Negative**  **Positive** | 351(70.1%)  150(29.9% | 366(77.7%)  105(22.3%) | **0.007** | 303(70.6%)  126(29.4%) | 381(75.9%)  121(24.1%) | 0.070 | 309(71.5%)  123(28.5%) | 366(75.0%)  122(25.0%) | NS |
| **BCL2**  **Negative**  **Positive** | 152(42.7%)  204(57.3%) | 98(27.5%)  258(72.5%) | **<0.001** | 158(46.6%)  181(53.4%) | 118(31.6%)  255(68.4%) | **<0.001** | 136(41.7%)  190(58.3%) | 138(36.6%)  239(63.4%) | NS |
|  | **Pan JNK1/2** | | | **p-JNK1/2** | | | **Pan P38** | | |
|  | **Neg/low** | **High** | **p-value** | **Neg/low** | **High** | **p-value** | **Neg/low** | **High** | **p-value** |
| **ER**  **Negative**  **Positive** | 87(18.4%)  386(81.6%) | 91(22.5%)  313(77.5%) | NS | 68(37.2%)  115(62.8%) | 130(19.0%)  553(81.0%) | **<0.001** | 1  57(26.6%)  434(73.4%) | 54(14.1%)  328(85.9%) | **<0.001** |
| **KI67-LI**  **Low**  **High** | 177(46.1%)  207(53.9%) | 151(47.9%)  164(52.1%) | NS | 51(34.0%)  99(66.0%) | 269(48.1%)  290(51.9%) | **0.002** | 207(42.4%)  281(57.6%) | 158(51.0%)  152(49.0%) | **0.018** |
| **P53**  **Negative**  **Positive** | 342(75.0%)  114(25.0%) | 293(74.6%)  100(25.4%) | NS | 127(71.3%)  51(28.7%) | 498(74.0%)  175(26.0%) | NS | 415(72.0%)  161(28.0%) | 294(78.0%)  83(22.0%) | **0.040** |
| **BCL2**  **Negative**  **Positive** | 118(34.1%)  228(65.9%) | 129(42.2%)  177(57.8%) | **0.034** | 67(48.2%)  72(51.8%) | 189(36.7%)  326(63.3%) | **0.014** | 172(41.4%)  243(58.6%) | 85(30.2%)  196(69.8%) | **0.003** |
|  | **p-p38** | | | **p-c-jun** | | | **p-ATF2** | | |
|  | **Neg/low** | **High** | **p-value** | **Neg/low** | **High** | **p-value** | **Neg/low** | **High** | **p-value** |
| **ER**  **Negative**  **Positive** | 179(23.2%)  593(76.8%) | 41(12.7%)  283(87.3%) | **<0.001** | 97(26.2%)  273(73.8%) | 116(17.1%)  563(82.9%) | **<0.001** | 176(22.9%)  594(77.1%) | 38(12.9%)  256(87.1%) | **<0.001** |
| **KI67-LI**  **Low**  **High** | 261(41.3%)  371(58.7%) | 148(57.8%)  108(42.2%) | **<0.001** | 111(37.1%)  188(62.9%) | 277(49.9%)  278(50.1%) | **<0.001** | 254(40.8%)  368(59.2%) | 138(56.3%)  107(43.7%) | **<0.001** |
| **P53**  **Negative**  **Positive** | 557(73.3%)  203(26.7%) | 242(76.8%)  73(23.2%) | NS | 260(71.8%)  102(28.2%) | 5  01(75.1%)  166(24.9%) | NS | 5  50(73.1%)  202(26.9%) | 226(77.7%)  65(22.3%) | NS |
| **BCL2**  **Negative**  **Positive** | 231(40.5%)  340(59.5%) | 67(28.4%)  169(71.6%) | **0.001** | 105(38.9%)  165(61.1%) | 186(37.2%)  314(62.8%) | NS | 221(38.4%)  354(61.6%) | 70(31.8%)  150(68.2%) | 0.083 |
| **Bold p-values are significant (<0.05), borderline p-values : 0.05-0.09, Non significant p-values (NS): >0.09** | | | | | | | | | |

|  | **a-**  **MCF7(ER+HER2-**  **/ MCF-Erb2(ER+HER2+)** | **b-**  **MCF7(ER+HER2-)**  **/BT474 (ER+HER2+)** | **c-**  **MCF7 (ER+HER2-)**  **/ MDA-231(ER-HER2-)** | **d-**  **MCF-Erb2(ER+HER2+)**  **/MDA-231 Erb2(ER- HER2+)** | **e-**  **MCF-Erb2 (ER+HER2+)**  **/SKBR3(ER-HER2+)** | **f-**  **MCF-Erb2 (ER+HER2+)**  **/MDA-231 Erb2 (ER-HER2+)** | **g-**  **BT474(ER+HER2+)**  **/SKBR3(ER-HER2+)** | **h-**  **MDA-231 (ER-HER2-)/**  **MDA-231 Erb2(ER-HER2+)** | **i-**  **MDA-231 (ER-HER2-)**  **/ SKBR3(ER-HER2+)** |
| --- | --- | --- | --- | --- | --- | --- | --- | --- | --- |
| **ERK1/2** | **<0.05** | **<0.05** | **<0.05** |  | **<0.05** |  | **<0.05** |  | **<0.05** |
| **pERK1/2** |  |  | **<0.05** |  |  |  |  | **<0.05** | **<0.05** |
| **P38** | **<0.05** |  | **<0.05** | **<0.05** | **<0.05** |  |  | **<0.05** | **<0.05** |
| **pP38** | **<0.05** |  | **<0.05** |  |  |  |  |  | **<0.05** |
| **pCRAF** | **<0.05** |  | **<0.05** | **<0.05** |  | **<0.05** |  |  | **<0.05** |
| **pMKK1/2** | **<0.05** | **<0.05** | **<0.05** | **<0.05** |  | **<0.05** |  | **<0.05** |  |
| **MKK7** |  |  |  |  | **<0.05** |  |  |  | **<0.05** |
| **MKK3** |  |  | **<0.05** |  |  |  |  |  |  |
| **MSK 2** |  |  |  | **<0.05** |  |  |  |  |  |
| **pSMAD3** | **<0.05** | **<0.05** |  |  |  |  |  | **<0.05** |  |
| **pATF2** | **<0.05** | **<0.05** | **<0.05** |  |  |  |  |  |  |
| **c-Jun (S63)** |  |  |  | **<0.05** |  | **<0.05** |  |  |  |
| **pc-Jun (S63)** |  |  |  | **<0.05** |  |  |  |  |  |
| **JNK1** |  |  |  |  |  |  |  |  |  |
| **pJNK1 (T183/Y185)** | **<0.05** | **<0.05** |  | **<0.05** | **<0.05** | **<0.05** | **<0.05** |  |  |
| **pELK1** | **<0.05** | **<0.05** |  |  |  |  |  |  |  |
| **pSTAT3** | **<0.05** | **<0.05** |  |  |  |  |  | **<0.05** |  |

**Table 6: Evaluation of the expression of MAPK signaling molecules in different BC cell lines:**

This table shows the obtained p values when comparing the levels of expression of 16 signaling markers. Significance values were derived using the Kruskal Wallis test (<0.5). a) ER+HER2- versus ER+HER2+(T), b) ER+HER2- versus ER+HER2+(W), c) ER+HER2-versus ER-HER2-, d) ER+HER2+(T) versus ER-HER2+(T), e) ER+HER2+(T) versus ER-HER2+(W), f) ER+HER2+(W) versus ER-HER2+(T), g) ER+HER2+(W) versus ER-HER2+(W), h) ER-HER2- versus ER-HER2+(T) and i) ER-HER2- versus ER-HER2+(W).


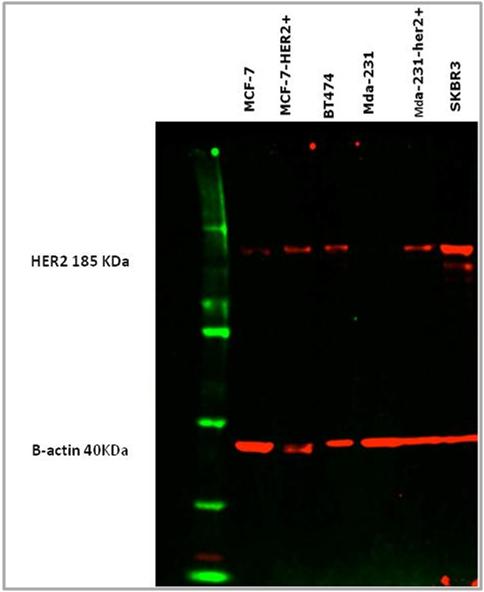


Fig 1 WB of HER2 protein in the six BC cell lines used in the current study


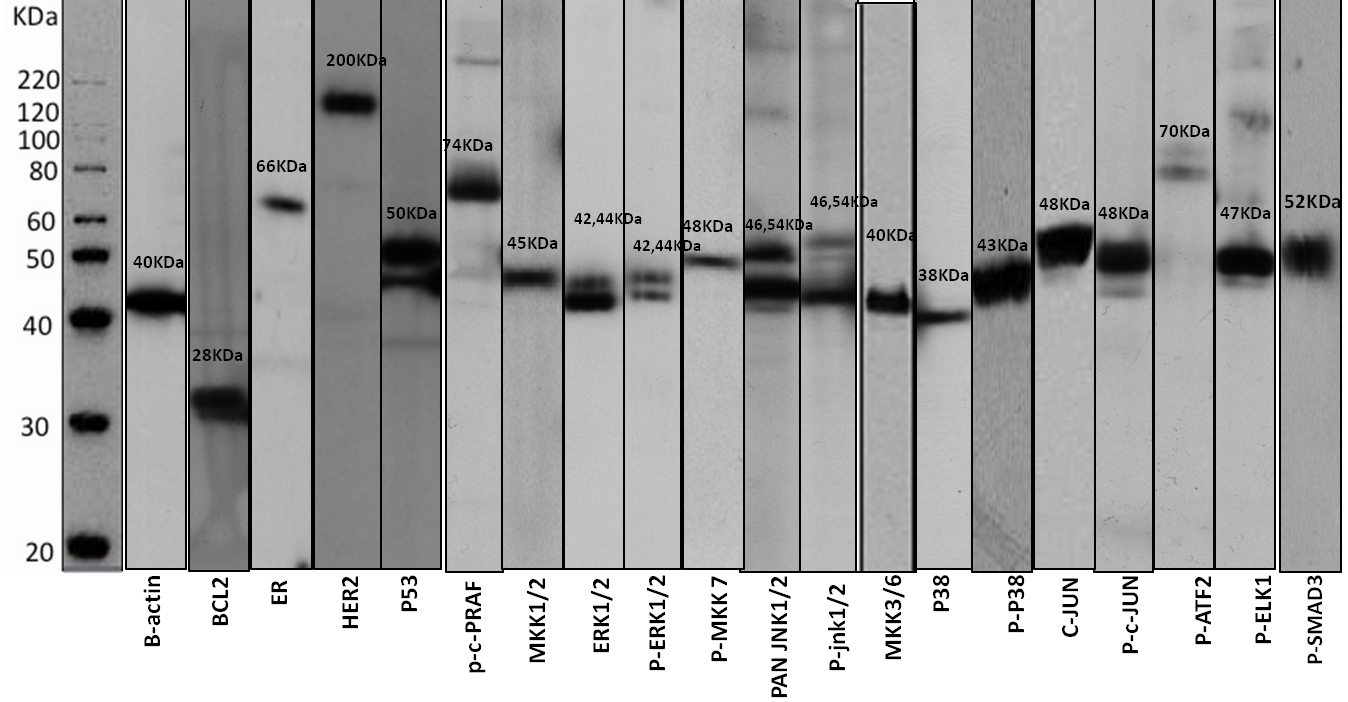


Fig 2 Western blot of biomarkers used in the current study


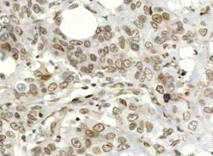

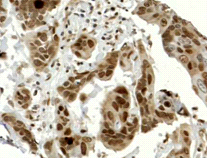

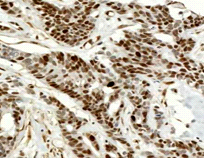

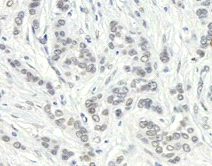

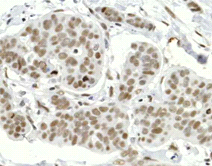

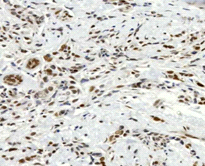

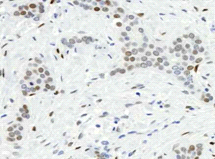

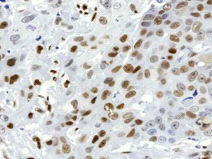

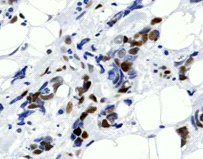


B

C

D

A


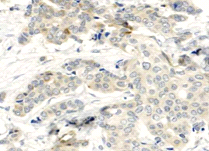

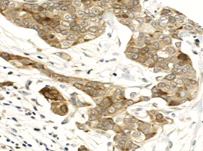

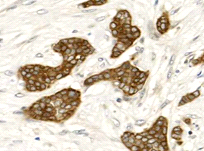


Fig 2 Western blot of biomarkers used in the current study

Fig 3 Different intensities of nuclear&/or cytoplasmic staining of MAPKs, from the left to right: Weak, moderate and strong intensities. A: pan p38, B: p-p38, C: p-ATF2 and D: p-C-JUN. All pictures were taken using digital pathology system at x20
